# Fabrication of PVA–Silica Sol Wood Composites via Delignification and Freezing Pretreatment

**DOI:** 10.3390/polym16131949

**Published:** 2024-07-08

**Authors:** Rizheng Cong, Taoyang Cai, Shangjie Ge-Zhang, Hong Yang, Chang Zhang

**Affiliations:** 1 School of Civil Engineering and Transportation, Northeast Forestry University, Harbin 150040, China; 2 Aulin College, Northeast Forestry University, Harbin 150040, China; 3 College of Science, Northeast Forestry University, Harbin 150040, China; 4 Research Institute of Intelligent Control and Systems, Harbin Institute of Technology, Harbin 150040, China

**Keywords:** delignification, freezing pretreatment, modified wood, compressive strength

## Abstract

The efficient exploitation of planted fast-growing wood is crucial for enhancing wood resource utilization. In this study, the fast-growing poplar wood was modified by in situ impregnation through vacuum impregnation with polyvinyl alcohol and nano-silica sol as impregnation modifiers, combined with delignification–freezing pretreatment. The samples were characterized by FTIR, XRD, SEM, and the universal mechanical testing machine. The results showed that the wrinkle deformation and cracking of the wood blocks were greatly alleviated after the delignification–freezing pretreatment and the polyvinyl alcohol and nano-silica sol were successfully integrated into the wood. The resulting polyvinyl alcohol–silica sol poplar composites exhibited about 216%, 80% and 43% higher compressive strength with respect to delignified wood, natural wood and impregnated natural wood, respectively, thereby demonstrating superior mechanical properties and potential opportunities for value-added and efficient utilization of low-quality wood.

## 1. Introduction

Forests, viewed as a natural, environmentally friendly and renewable resource, serve an invaluable role in the global carbon cycles. Apart from assisting in hydrological regulation and soil sequestration, trees continue to sequester carbon throughout their life spans, which is also a key component of climate change mitigation efforts to achieve carbon neutrality targets [1,2,3,4]. Nevertheless, the rapid expansion of society is leading to an increasing consumption of wood, and it has emerged as a pressing issue to balance harvesting and carbon sequestration for sustainable development [5]. Although fast-growing wood offers new opportunities for maintaining high wood volumes without disrupting the carbon balance in contrast to traditional wood, its short growth cycle inevitably brings about disadvantages, such as looseness of material, inferior mechanical strength and insufficient durability, which severely limits the service life of its products and restricts its large-scale application.

The delignification treatment enables the removal of lignin and hemicellulose from the wood, which on the one hand creates more channels and anchor points inside the wood, while on the other hand alters parts of the physical and mechanical properties of the wood, thus exerting an influence on the prepared composites. The alkaline delignification process selectively dissolves lignin from the wood cell walls, increasing the accessible surface area and accelerating the dissolution of lignin, among other behaviors, thereby triggering modifications in the microstructure and macroscopic properties of the wood [6,7]. However, excessive delignification may cause crinkling and splitting of the wood. The crinkling refers to the deformation of the wood resulting from the movement of moisture during drying, inducing uneven surfaces, irregular cross-sections and even shrinkage of the wood, which in turn may lead to an increase in the rate of damage to the wood product, to the detriment of further processing and subsequent application [8]. The cryogenic treatment is remarkable for its effectiveness in enhancing the structural properties and stability of the material, as well as being described as an environmentally friendly physical treatment. With the proven low-temperature pre-freezing treatment, the water in the cell cavities of the process freezes into ice, destroying microstructures such as the grain pore membrane and improving the permeability of the wood. In addition, at lower temperatures, the ice inside the wood sublimates directly into water vapor without generating liquid capillary tension, which can effectively prevent wrinkling and collapse of the wood and improve the wood properties, which can be of great assistance in subsequent comprehensive wood modification [9,10].

For further advancement of mechanical properties, researchers typically compress wood after delignification or fill it with other materials to obtain enhanced mechanical properties [11,12,13,14,15,16]. Among these, impregnation treatment is a commonly employed green and facile method for wood modification [17,18]. More recently, the introduction of inorganic nanoparticles into organic polymers has gained much attention, and organic–inorganic nanocomposites offer an effective approach to optimize decay resistance, physical and mechanical properties, UV stability, and so on [19,20,21]. Dong et al. prepared a polymer–SiO_2_ hybrid nanocomposite solution by the sol–gel method. The wood impregnated with this composite solution has excellent compressive strength and has the potential to be used as a substitute for wood–plastic composites and high-quality solid wood materials [22]. Chang et al. used silicon dioxide–polymer hybrid material, TEOS as an inorganic precursor and HDTMS as an organic modifier to prepare wood superhydrophobic coating by sol–gel chemistry, and it was still stable in strong acid and alkali environment [23]. Hoyos-Martínez et al. successfully prepared wood fire-retardant coatings based on the bio-based phenolic resin formula of lignin, tannin and inorganic nanoparticles [24].

In this study, a PVA–silica sol-modified impregnation solution was prepared, and the poplar fast-growing wood was modified in situ by delignification–freezing pretreatment and vacuum impregnation. This method is simple and economical. The effects of the impregnation solution on the delignification–freezing treatment of samples were investigated, and the effects of the delignification–freezing treatment method and PVA–silica sol-modified impregnation solution on the chemical structure, crystallinity properties and mechanical properties of poplar fast-growing wood were discussed. This method improved the impregnation rate and obtained high-strength wood with better mechanical properties.

## 2. Materials and Methods

### 2.1. Materials

The poplar blocks (Populus ussuriensis Kom, 21 years old) were collected from a plantation forestry farm in Shijiazhuang, Hebei Province, China. The samples were extracted from wood that presented no knots, discoloration, fungi or other obvious flaws and were cut to the dimensions of 30 mm × 20 mm × 20 mm (L × T × R). All blocks were continuously sanded with 600–2000-grit metallographic sandpaper until the dust was completely removed and were subsequently stored at 20 °C and 65% relative humidity to achieve hygroscopic equilibrium. Anhydrous ethanol (99%), NaOH (>96%), Na_2_SO_3_ (>97%) and deionized water were supplied by Harbin Junan Medical Glass Wholesale Station; polyvinyl alcohol (PVA, Mw~27,000) was purchased from Maclean’s Ltd., Shanghai, China; and silica sol (JN-30) was acquired from Shandong Youso Chemical Technology Co., Ltd., Linyi, China. All of the chemical raw materials were analytically pure and did not require further purification for use.

### 2.2. Delignification–Freezing Pretreatment of the Poplar Wood Blocks

An aqueous solution of 1.25 mol·L^−1^ NaOH and 0.4 mol·L^−1^ Na_2_SO_3_ was prepared at room temperature and magnetically stirred for 10 min to obtain a delignification solution. The poplar samples were immersed in the delignification solution and placed in a constant temperature water bath shaker and hydrothermally treated at 95 °C for 6 h under 35 rpm. The above procedure was performed to remove the lignin and hemicellulose from the wood. The samples were picked up and washed several times with boiling deionized water until the solution was almost colorless. The cleaned delignified wood was stored at −30 °C for 12 h and then dried to a steady weight in a vacuum drying oven to obtain delignified–frozen treated wood, referred to as DW.

### 2.3. Preparation of PVA–Silica Sol-Modified Impregnating Solution

The PVA powders were dissolved in deionized water and stirred for 6 h at 90 °C with a collector-type thermostatic heating magnetic stirrer (Yuhua DF-101S, Gongyi City Yuhua Instrument Co., Ltd., Gongyi, China) to obtain the aqueous PVA solution of 3wt%. The aqueous PVA solution and silica sol were then mixed in a mass ratio of 3:1 and ultrasonicated for 30 min with an ultrasonic cleaner (Fuyang F-020SD, Shenzhen Fuyang Technology Group Co., Ltd., Shenzhen, China) at a power of 180 W, followed by magnetic stirring at room temperature for 6 h to yield a homogeneous and stable PVA–silica sol-modified impregnation solution.

### 2.4. Preparation of PVA–Silica Sol-Modified Wood Based on Delignified Lignin Wood Framework

**Figure 1:**
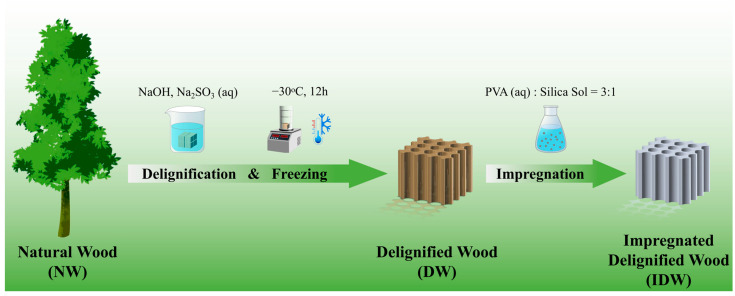
Schematic of the fabrication of composite wood.

The dried DW samples were infiltrated into the PVA–silica sol-modified impregnating solution under vacuum for 2 h and then kept at room temperature and pressure for 22 h for adequate soaking. After the completion of impregnation, the poplar samples were removed and the residual mixture solution on the surface was washed several times with deionized water, drained at room temperature and subsequently dried and cured under a vacuum at 50 °C for 24 h to obtain PVA–silica sol poplar composites (impregnated delignified wood, IDW). A detailed preparation schematic is shown in Figure 1.

### 2.5. Characterization

The surface morphological features of the wood samples were characterized with a cold field emission scanning electron microscope (FE-SEM, JSM-7500F, JEOL Ltd., Akishima, Japan), and the chemical elements in the samples were examined by an associated Oxford X-Max cooling energy spectrometer. The wood was cut into small slices parallel to the direction of growth, i.e., radial sections, using a pathological sectioning blade, and the cross-sectional micromorphology of NW and IDW slices at an accelerating voltage of 5.0 kV was analyzed by FE-SEM, and the homogeneous distribution of the modifier in the samples was further verified by the chemical elemental composition as tested by EDS [25,26,27]. The samples were ground into 200–300 mesh powders and assessed for the variations in the chemical composition of the wood using Fourier transform infrared spectroscopy (FTIR, Nicolet iS50, Thermo Fisher Scientific Inc., Waltham, MA, USA) with 32 scans in the wavelength range of 3000–400 cm^−1^ at a resolution of 4 cm^−1^. The samples were scanned using an X-ray diffractometer (XRD, XRD-6100, Shimadzu Corporation, Kyoto, Japan), which was carried out to inspect the crystal structure by scanning samples ground to 200–300 mesh. The radiation source voltage was set at 40 kV, the radiation tube current at 30 mA, and the scanning range and measurement rate at 5–85° and 10°/min, respectively. Compression tests were conducted along the grain direction using a universal mechanical testing machine (Sanshi CMT-6305, Shenzhen, China), following the guidelines of reference standard GB/T 1935-2009 [28]. Prior to testing, samples were conditioned in a chamber set at a constant temperature of 20 °C and relative humidity of 65% to ensure uniform moisture content. Testing was carried out at room temperature (20 ± 5 °C), with a loading speed of 10 mm/min. The compression strength of five samples was calculated, and the average value was considered as the mean compression strength. The thermogravimetric analysis of the sample was carried out by using a simulated thermal analyzer (STA, Perkin Elmer STA 6000, Waltham, MA, USA), and the mass loss of the sample was measured at 30 °C to 800 °C at a heating rate of 10 °C min^-1^. Hygroscopicity was evaluated according to GB/T 1934.1-2009 [29]; dry shrinkage also uses this standard. In addition, in order to evaluate the wettability of the composites, the static water contact angle of the samples was measured by a droplet shape analyzer (DSA100, Krüss, Hamburg, Germany), and the droplet volume of 5 μL was selected.

## 3. Results and Discussion

### 3.1. Impregnation Rate and Weight Increase Rate

The weighing was conducted using an analytical balance. The mass of the dried sample before impregnation is recorded as m_0_ and the mass of the sample after impregnation as m_1_. The impregnated sample is dried in a vacuum drying oven at 50 °C to a constant weight and then quickly removed and weighed to prevent the wood from absorbing moisture, and the mass of the impregnated sample after drying is recorded as m_2_. The rate of impregnation and the rate of weight increase for NW and DW are calculated and compared according to the following two equations:

(1) $$\begin{array}{c} \mathrm{Impregnation}\;\mathrm{Rate}=\frac{m_{1}-m_{0}}{m_{0}}\times 100\% \end{array}$$

(2) $$\begin{array}{c} \mathrm{Weight}\;\mathrm{Increase}\;\mathrm{Rate}=\frac{m_{2}-m_{0}}{m_{0}}\times 100\% \end{array}$$

**Figure 2:**
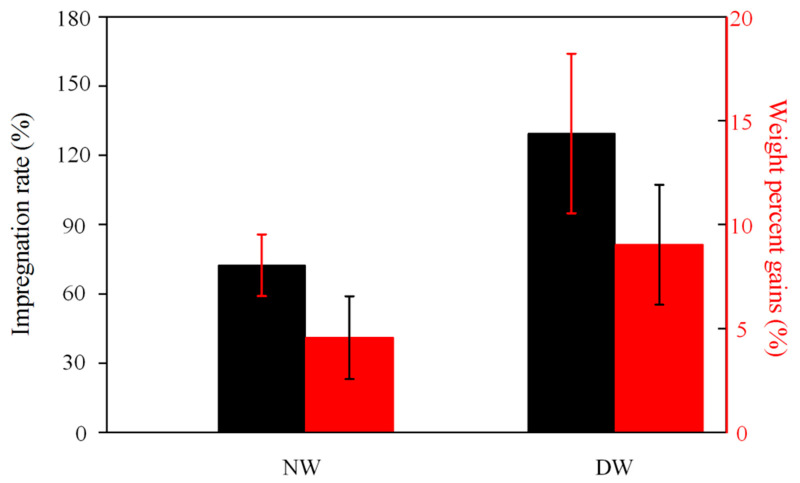
Impregnation and weight gain rates of NW and DW.

Since it is known that the presence or absence of lignin significantly affects the modification effect, an excessive lignin content weakens the modification effect [30,31]. After the delignification of the samples using a mixture of NaOH and Na_2_SO_3_ solutions, most of the lignin and hemicellulose of the wood was removed, which will be confirmed in the FTIR and XRD patterns below [32], and it increased the porosity of the wood and provided more channels and sites for the impregnation of the modified impregnating solution, which will further improve impregnation effect [33,34]. The impregnation effect can be visualized by calculating the impregnation rate and the weight gain rate. Figure 2 shows the impregnation and weight gain rates for NW and DW, where the black bars show a 78.20% increase in impregnation rate for delignified–frozen treated wood (DW) compared to direct impregnation with NW, and the red bars show a 98.03% increase in weight gain for DW compared to NW. This indicates that the degree of impregnation of the delignin–frozen treated samples was increased and that the added modified impregnating solution was effective in cross-linking with the wood [35], resulting in an increase in weight gain.

### 3.2. Micromorphology and Microstructure

**Figure 3:**
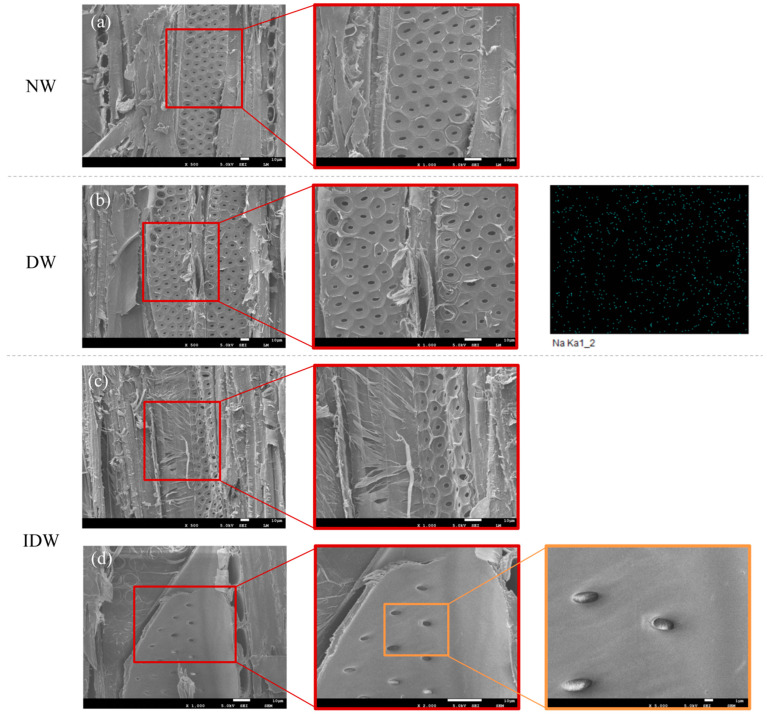
The SEM images and EDS images of NW (**a**), DW (**b**), IDW (**c**,**d**) at different magnifications.

The FE-SEM equipped with EDS enabled us to determine the microstructural morphology and elemental distribution of the samples more precisely. The EDS images in Figure 3b show that, despite repeated washing with boiling deionized water, a small amount of Na from the delignification solution remained uniformly on the surface of the wood, (3.27%), i.e., the wood was uniformly and profoundly delignified. By comparing the SEM images of the untreated NW radial section in Figure 3a and the DW radial section in Figure 3b, it is clear that under the action of the delignification solution, some components of wood are selectively removed, exhibiting larger and more irregular lumens and higher porosity [36,37], which provides more channels for the penetration of the PVA–silica sol-modified impregnating solution in the subsequent steps. In addition, the porous and layered structure of the poplar samples was not significantly affected by the delignification–freezing treatment, as the pre-freezing treatment resulted in “cold shrinkage” due to the loss of water from the cell walls, and the rapid sublimation of the ice prevented fluid movement and reduced macroscopic wrinkling of the wood, in addition to the fact that the expanding ice disrupted part of the microstructure of the wood, improving the permeability of the wood. In addition, the expanding ice disrupts part of the microstructure of the wood, improving the permeability of the wood and making subsequent PVA–nano-silica sols easier to infiltrate [38,39]. Figure 3c is an SEM image of the IDW, demonstrating the uniform distribution of the PVA–silica sol-modified impregnation on the wood cell walls, with no tight gaps between them. Further, as shown in Figure 3d, the filling of the grain pores with the impregnation solution can be clearly observed in the SEM image of the IDW sample at a magnification of 5000, indicating that the impregnation of the DW was adequate and complete.

### 3.3. Chemical Composition

**Figure 4:**
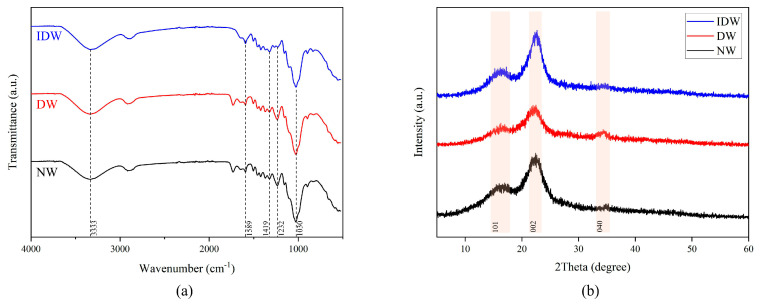
FTIR (**a**) and XRD (**b**) patterns of NW, DW, IDW.

The samples from the NW, DW and IDW groups were analyzed by FTIR spectroscopy to investigate the effect of delignification–freezing treatment versus modified impregnation solution on the chemical structure of poplar wood. Figure 4a illustrates the FTIR spectra of the three samples, all of which reveal an absorption vibration peak of -OH around 3333 cm^−1^, a phenolic hydroxyl vibration peak of lignin at 1419 cm^−1^ [40] and a strong characteristic absorption peak of C-O at 1030 cm^−1^. The samples from the DW and IDW groups show significantly enhanced -OH absorption vibrations around 3333 cm^−1^ compared to NW, which is partly due to the increased content of bound hydroxyl groups in the impregnated modified wood due to the high hydrophilic hydroxyl group of PVA itself, and partly due to the formation of more hydrogen bonds between the modified impregnate and the wood. The poplar samples from the IDW group display a weakening of the C=O stretching vibration peak at 1734 cm^−1^. The fact that the activation energy of delignified wood is always less than that of untreated wood suggests that the C=O double bonds in the delignified wood-modified wood are more likely to break and are more likely to undergo additional reactions with the silica nanosol. As can be seen in Figure 4a, the characteristic Si-O-Si vibrational peak appears at 1589 cm^−1^ and the enhancement of the Si-O-Si vibrational peak is more pronounced for IDW. This suggests that delignin promotes the chemical reaction of the PVA–silica sol to form a cross-linked system within the wood, with increasingly larger molecules but fewer hydrophilic Si-O- groups, rendering the molecules insoluble in water and improving the hydrophobicity of the composite wood [37,38,39,40].

The variation in the crystalline structure of cellulose in the cell walls of wood affects the size of its crystalline zones, cell parameters and crystallinity. The relative crystallinity of wood is basically the percentage of the cellulose crystalline zone in relation to the total cellulose. Variations in crystallinity are closely related to the dimensional stability, hardness, tensile strength and density of the fibers of the wood. According to the two-phase system theory of cellulose structure, in the crystalline region, the cellulose molecular chains are arranged in a directionally ordered manner, whereas in the non-crystalline region, they are irregularly arranged [41], and X-ray diffraction allows for the detection of highly crystalline cellulose molecular chains [42]. To investigate the effect of the hybridized system formed by delignification–freezing treatment and PVA–nano-silica sol on the crystal structure of the modified wood, the NW, DW and IDW groups were examined using XRD, and the results are displayed in Figure 4b. In the X-ray diffraction pattern of the wood, the highest peak is located at 2θ = 22.42°, which is the 002 crystal plane and represents the width of the crystalline region. At 2θ = 34.7°, which is the 040 crystal plane, represents the length of the crystal zone. The 002 crystal plane has a higher intensity than the 040 crystal plane due to the molecules within the cellulose being located mainly in planes parallel to the 002 crystal plane. A 101 wave is observed at 2θ = 16.5°, which is the diffraction intensity of the amorphous region. A comparison of the three sets of spectra shows no significant change in the position of the diffraction peaks, i.e., the cellulose crystalline regions of NW, DW and IDW have not suffered alteration [43]. Delignin breaks the hydrogen bonds of the cellulose itself weakening the inter-molecular interactions between the cellulose molecular chains and reducing the crystallinity [44]. In general, the crystallinity of wood is positively correlated with the mechanical strength of wood [45]. Although the crystallinity of delignified DW was lower and the mechanical properties were weaker, the compressive strength of the IDW group was higher than the other groups in the mechanical property tests, which may be due to the reaction of PVA with wood cellulose after it has been immersed inside the wood to create stronger chemical bonds and improve the mechanical properties.

In summary, based on SEM-EDS images, FTIR and XRD patterns, we can infer that delignification and freezing treatments give more possibilities for PVA and silica sol to incorporate into the wood, cross-linking and filling the pores of the wood cell walls, thus ameliorating the properties of the wood.

### 3.4. Physical and Mechanical Properties

**Figure 5:**
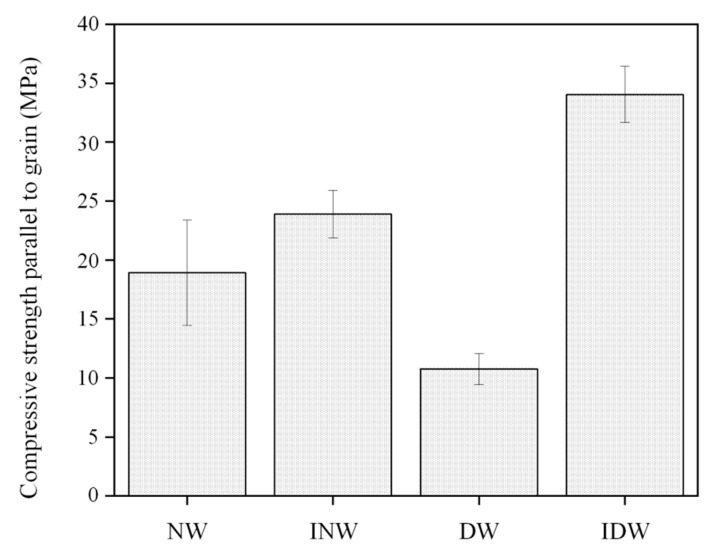
Mechanical properties of several groups of poplar wood samples.

The compressive strength parallel to the grain means the maximum stress produced by pressure in the direction of the grain and is one of the most important indicators of the mechanical properties of wood. The mechanical properties of the NW, INW, DW and IDW groups were tested in Figure 5. The data are derived from the mean value of the compressive strength of five randomly selected samples in each group. As shown in Figure 5, the smooth grain compressive strengths of the DW, NW, INW and IDW groups increased in order. Compared to the NW group, the average compressive strength of the samples from the INW and IDW groups increased by 26% and 80%, respectively, indicating that the filling of the PVA–silica sol-modified impregnation could increase the compressive strength of the wood [46,47]. Although the DW group, which was only delignified–chilled, showed a sharp decrease in compressive strength due to the reduction of lignin and hemicellulose and the disappearance of the wood’s internal support material, the IDW group obtained by impregnating it with PVA–silica sol was the strongest, still 43% higher than the INW group, which had the second strongest compressive strength, due to the fact that the delignification treatment provided more channels inside the wood and space within the wood, allowing the PVA–nano-silica sol to fill the pores and lamellar structures in the poplar wood more fully and more often. On the other hand, the pre-freezing treatment improves the permeability of the wood, making it easier for the PVA–nano-silica sol to infiltrate. During the impregnation process, as the modified impregnation solution is cross-linked with PVA and nano-silica sol, which has a large number of hydroxyl groups on the outside, the delignified wood is also able to expose more hydroxyl groups and therefore can form hydrogen bonds more tightly, thus increasing the compressive strength of the IDW. Compared with the literature data of similar systems, the samples prepared in this study have good mechanical properties [33,48,49].

### 3.5. Water Contact Angle, Hygroscopicity and Dry Shrinkage

**Figure 6:**
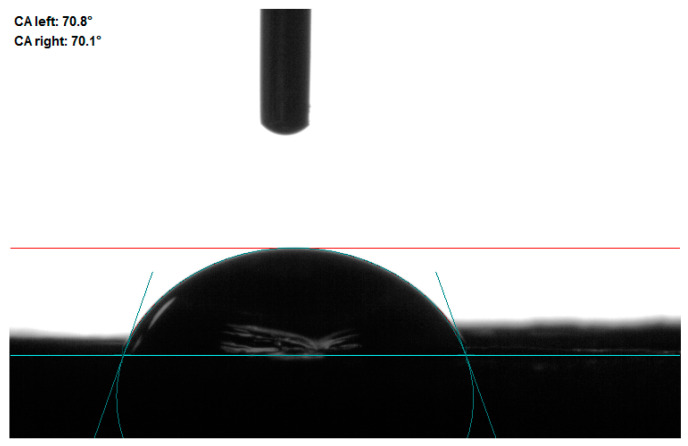
Static water contact angle of IDW surface.

Static wetting behavior was evaluated using 5 microliters of droplets. NW and DW were completely absorbed within 10 s and 2 s after the droplets contacted the surface (Videos S1 and S2), respectively, showing strong hydrophilicity. The faster water absorption of DW may be due to delignification, which removes most hydrophobic lignin, exposes more hydroxyl groups and increases the porosity of wood. IDW tends to be stable after the droplet contacts the surface for 6 s, and finally, a static contact angle of 70° is obtained (Figure 6, Video S3); the improvement in hydrophobicity is mainly attributed to the increase in surface roughness and the barrier of surface film to water [50]. The silica nanoparticles on the wood surface will affect the wood surface roughness and capture more air, and PVA will coat the nanoparticles and form a film on the surface of delignified wood to slow down the water infiltration. In future experiments, it is necessary to use low-surface energy substances to modify the surface in order to further realize hydrophobicity [51,52,53,54].

The water absorption and dry shrinkage of DW and IDW are given in Table 1. Due to its rich pores and hydrophilicity, DW shows strong hygroscopicity, and its 24-h water absorption rate reaches 96%, while IDW is ~68%. After 2 weeks of continuous water absorption, the water absorption rate of DW is basically maintained at around 155%, while the IDW is still below 105%. PVA impregnation can effectively reduce the water absorption of wood, which may be due to the fact that PVA will form a film inside the wood during impregnation, blocking these pores, thus reducing the passage of water into the wood [55]. In addition, PVA impregnation can also reduce the water absorption by enhancing the structural stability of wood and reducing moisture absorption and expansion. For drying shrinkage, the radial full drying shrinkage of NW is 5.7%; the tangential full drying shrinkage is 7.1%. The shrinkage rate of volume full drying is 12.8%. Compared with NW, the radial shrinkage of IDW is slightly increased, while the tangential shrinkage and volume shrinkage are slightly decreased, which are 6.2%, 6.9% and 12.8%, respectively. It is speculated that PVA may play a role in filling and enhancing the structural stability of wood and reducing the deformation of wood in the tangential direction [56].

### 3.6. Thermogravimetric Properties

**Figure 7:**
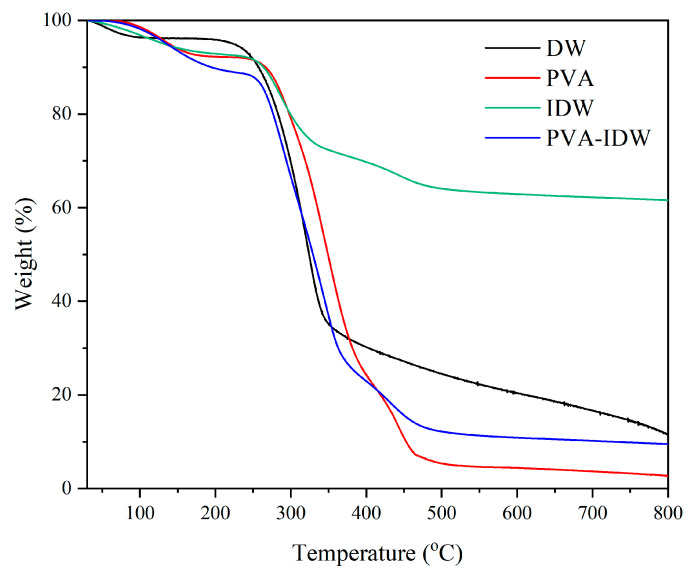
A typical TGA thermogram of DW, pure PVA, IDW and PVA-IDW.

The thermal stability of various samples between 30 °C and 800 °C was investigated using thermogravimetric analysis (Figure 7). For delignified wood (DW), the initial mass loss from room temperature to 105 °C is attributed to water evaporation (~4%). A negligible weight loss is observed between 105 °C and 220 °C, during which the sample absorbs heat slowly, resulting in minimal mass change and a low weight loss rate. The highest weight loss rate, approximately 60%, occurs during the pyrolysis stage between 220 °C and 350 °C, due to the initial oxidative decomposition of hemicellulose and cellulose, as most lignin has been removed from DW. Hemicellulose begins to pyrolyze at 220 °C, with substantial decomposition products forming between 250 °C and 350 °C. Beyond 350 °C, the degradation rate decreases significantly, entering the carbonization stage where residual substances slowly pyrolyze until carbonization, resulting in little mass change. Post-experiment, only black carbon remains in the crucible. Pure PVA exhibits three distinct weight loss stages in its thermogravimetric analysis. From room temperature to 150 °C, the first stage shows a gentle downward trend due to the loss of physical water, with a mass loss of approximately 7%. The second stage, between 260 °C and 380 °C, shows a mass loss of up to 60%, primarily due to dehydration caused by the elimination of hydroxyl side groups. The final stage, from 400 °C to 460 °C, corresponds to the breakdown of the main PVA chain, leaving a low residual mass characteristic of polymeric materials [57]. The PVA-IDW sample, impregnated only with pure PVA, demonstrates overlapping degradation stages of cellulose and PVA from 200 °C to 450 °C, with the final mass residue falling between those of DW and pure PVA. Notably, the residual mass fraction of IDW at 800 °C is approximately 61%, significantly higher than that of DW and PVA-IDW, indicating enhanced thermal resistance. This improvement may be due to the excellent thermal properties of nano-silica, and with the increase in temperature, nano-silica remains stable without mass loss.

## 4. Conclusions

In this paper, the fast-growing poplar wood is modified under vacuum impregnation using PVA–silica sol as an impregnation modifier, in combination with delignification–freezing treatment. The prepared PVA–silica sol poplar wood composites possess excellent smooth grain compressive strength, and the impregnation effect, structural modifications and compressive strength were investigated for analysis. The results showed that the removal of lignin and the increase in porosity in the delignified poplar samples facilitate the entry of PVA and silica sol into the wood, which is macroscopically expressed as an increase in weight gain and impregnation rate of IDW, and an 80.01% increase in compressive strength compared to NW. In summary, the method proposed in this study provides a research idea for the high-value utilization of low-quality wood and offers new possibilities for the application of wood in the construction industry. Additionally, given the unique adsorption characteristics of nano-silica, a promising future research direction involves modifying the silica before impregnation (such as introducing silver nanoparticles or natural antibacterial components) to enhance its antibacterial properties [58,59].

## Figures and Tables

**Table 1:** Water absorption and full dry shrinkage of DW and IDW.

		NW	IDW
**Water absorption (%)**	1 day	96	68
	3 day	121	83
	7 day	142	94
	10 day	148	99
	12 day	152	101
	14 day	155	103
**Full Dry Shrinkage (%)**	Radial	5.7	6.2
	Tangential	7.1	6.9
	Volume	12.8	12.8

## Data Availability

The original contributions presented in the study are included in the article/Supplementary Materials, further inquiries can be directed to the corresponding author/s.

## Supplementary Materials

The following supporting information can be downloaded at https://www.mdpi.com/article/10.3390/polym16131949/s1. Video S1: Absorption time of droplets after contacting NW surface, Video S2: Absorption time of droplets after contacting the DW surface, Video S3: Absorption time of droplets after contacting the IDW surface.
